# Two cases of successful sirolimus treatment for patients with activated phosphoinositide 3-kinase δ syndrome 1

**DOI:** 10.1186/s13223-023-00840-0

**Published:** 2023-09-23

**Authors:** Lu Jiang, Xiaohan Hu, Qiang Lin, Ruyue Chen, Yunyan Shen, Yun Zhu, Qinying Xu, Xiaozhong Li

**Affiliations:** 1 Department of Nephrology and Immunology, Children’s Hospital of Soochow University, No. 303, Jingde Road, Suzhou, 215003 Jiangsu China; 2 Institute of Pediatrics, Children’s Hospital of Soochow University, Suzhou, 215003 China

**Keywords:** Activated PI3Kδ syndrome, PIK3CD, Genetic variant, Sirolimus

## Abstract

**Background:**

Activated phosphoinositide3-kinase (PI3K) δ syndrome 1 (APDS1) is a novel inborn errors of immunity (IEIs) caused by heterozygous gain of function mutations in PI3Kδ catalytic p110δ (PIK3CD). APDS1 has a spectrum of clinical manifestations. Recurrent respiratory infections, lymphoproliferation, hepatosplenomegaly, hyper-IgM syndrome and autoimmunity are the common symptoms of this disease.

**Case Presentation:**

Patient 1 presented with recurrent respiratory infections, hepatosplenomegaly and hyper-IgM syndrome. Patient 2 developed early onset systemic lupus erythematosus (SLE)-like disease with resistant thrombocytopenia. c.3061 G > A and c.2314G > A variants in the PIK3CD gene were detected by whole exome sequencing in two patients respectively. c.2314G > A variant in PIK3CD gene of patient 2 is a newly report. After genetic diagnosis, two patients received sirolimus treatment and sirolimus alleviated clinical manifestations, including hepatosplenomegaly in patient 1 and thrombocytopenia in patient 2.

**Conclusion:**

Genetics diagnosis should be considered in patients with complicated clinical manifestations with no or insufficient response to the conventional therapies. If whole exome sequencing suggests a variant in PIK3CD gene, sirolimus may relieve hepatosplenomegaly and resistant thrombocytopenia. This is the first report of c.2314G > A variant in PIK3CD gene.

**Supplementary Information:**

The online version contains supplementary material available at 10.1186/s13223-023-00840-0.

## Background

Human inborn errors of immunity (IEIs) are rare diseases caused by damaging genetic variants. IEI can be dominant or recessive, autosomal or X-linked inheritance, and the clinical phenotype can be complete or incomplete penetrance. The main clinical manifestations of these rare diseases are susceptibility to infection, as well as combining with autoimmune, autoinflammatory, allergic and/or malignant diseases. They now comprise 485 genetic disorders listed in the 2022 International Union of Immunological Societies (IUIS) classical classification [1].

Activated phosphoinositide3-kinase-δ (PI3Kδ) syndrome (APDS) is a recently reported IEI. PI3K molecules are composed of a p110 catalytic subunit and a regulatory subunit. Autosomal dominant gain-of-function (GOF) mutation in the PIK3CD gene encoding the PI3Kδ catalytic subunit p110δ cause APDS1, whereas autosomal dominant loss-of-function (LOF) mutation in the PIK3R1 gene encoding the regulatory subunit p85α cause APDS2 [2]. The hyperactivation of PI3Kδ results in the activation of the AKT-mTOR pathway, which further phosphorifies AKT and ribosomal S6 kinases in lymphocytes. The hyperactivated PI3K-AKT-mTOR pathway leads to functional defects of B lymphocytes, T lymphocytes and natural killer (NK) cells [3–5]. APDS1 patients develop a wide spectrum of clinical phenotypes, including recurrent respiratory tract infections, chronic epstein barr virus (EBV) and/or cytomegalovirus (CMV) viremia, benign lymphoproliferation, increased risk of lymphoma and autoimmunity [6]. Immunological features of these patients include low/normal serum level of immunoglobulin A (IgA) and IgG, normal or increased IgM [7], reduced class-switched memory B cells and CD4^+^ lymphopenia [8].

To date, 12 different activating missense mutations of PIK3CD gene have been revealed in APDS1, such as c.3061G > A, c.1002C > A, c.1246 T > C, c.1573G > A, and et al., of which c.3061G > A appears to be the most prevalent, accounting for approximately 85% [9–13]. Herein, we presented the clinical features and genotypes of two pediatric patients to show the diversity of APDS1 and to discuss the effect of sirolimus in curing APDS1. Especially, a novel variant in PIK3CD was reported.

### Case presentation

**Fig. 1:**
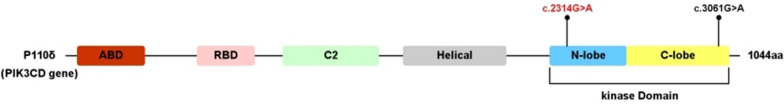
The locations of the variants in the PIK3CD gene. Black site is a hot spot variant in the PIK3CD gene of patient 1, and red site is a new variant in the PIK3CD gene of patient 2

Patient 1 was a 1 year and 5 months old male that presented with hepatosplenomegaly for more than one year and vomiting and diarrhea for one week. The medical history included recurrent respiratory tract infections, but no recurrent abdominal pain or diarrhea. When admitted to our hospital, the patient weighed 8.5 kg (< 3rd percentile) and had enlarged axilla lymph nodes. Abdominal ultrasonography showed hepatosplenomegaly, such that the liver was 2 cm below the right subcostal and the spleen was 3 cm below the umbilicus. Complete blood count values were as follows: white blood cells (WBC) 6.27 × 10^9^/L (normal, 4–10 × 10^9^/L), hemoglobin (Hb) 109 g/L (normal, 110–140 g/L), platelets (PLT) 76 × 10^9^/L (normal, 85–303 × 10^9^/L), and C-reactive protein (CRP) 3.04 mg/L (normal, 0–8 mg/L). Immunological values were as follows: CD3 919/μl (normal, 744–879/μl), CD4 422/μl (normal, 349–469/μl), CD8 496/μl (normal, 240–341/μl), CD4/CD8 0.9 (normal, 1.0–1.9), and CD19 61/μl (normal, 85–211/μl). Serum IgG, A, and M levels were 3.75 g/L (normal, 3.82–10.58 g/L), 0.8 g/L (normal, 0.14–1.14 g/L), and 1.59 g/L (normal, 0.4–1.28 g/L), respectively (Table 1). The EBV and CMV DNA load in the plasma were negative. Bone marrow biopsy excluded the possibility of malignancy. Computed tomography (CT) findings showed enlargement of multiple axilla nodes and hepatosplenomegaly. The whole exome sequencing of venous blood was performed on the child and his parents. A heterozygous missense mutation c.3061G > A (p.E1021K, Fig. 1, Table 2) of PIK3CD gene was revealed in this patient and his mother. His mother has not any clinical symptoms. After treatment with piperacillin and glucocorticoids for one week, symptoms of vomiting and diarrhea improved, but hepatosplenomegaly persisted. Subsequently, sirolimus (1 mg/m2) was started and sirolimus levels were maintained around 5.6–6.2 ng/mL. During follow-up, diarrheal episodes decreased, body weight increased, and hepatosplenomegaly significantly improved. However, the low IgG level and high IgM level have not fully recovered. Other serious adverse events were not noted.

**Table 1:** Clinical phenotypes and gene mutation sites in two patients

Age at genetic diagnosis/sex	Patient 1 (1 year, male)	Patient 2 (4 year, male)
Respiratory infections	√	√
Hepatomegaly	√	N
Splenomegaly	√	N
Lymphadenectasis	√	N
Thrombocytopenia	N	√
ANA	−	+
dsDNA	−	+
Immunoglobulins	IgM ↑ IgG ↓ IgA nl	IgM ↑ IgG ↑ IgA ↑
Complement	nl	C3 ↓ C4 ↓
Mutation site	c.3061 G > A	c.2314G > A
New mutation site	N	√
Diagnosis	APDS1	SLE/APDS1

√, present; + , positive; −, negative; ↑, increased abundance; ↓, decreased abundance; nl, normal; N, none

**Table 2:** PIK3CD missense mutations identified in the two patients

	Patient 1	Patient 2
Nucleotide change	c.3061 G > A	c.2314G > A
Amino acid change	p.E1021K	p.G772S
Exon	24	18
ExAC	−	0.0002
gnomAD_exome	−	0.0002
SIFT	Damaging	Tolerated
PolyPhen	Possibly damaging	Benign
GERP + +	Conserved	Nonconserved
Mutation Taster	Disease causing	Polymorphism
CADD phred	24.7	11.82
ACMG Classification	Pathogenic	Likely benign

**Fig. 2:**
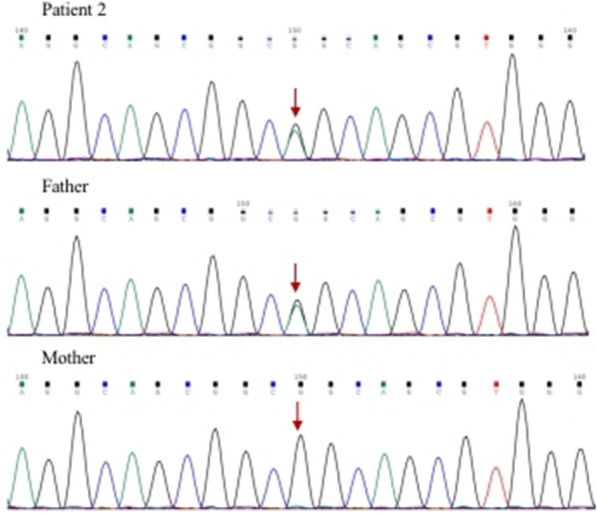
Sanger sequencing images of the patient 2 and his parents. Sanger sequencing chromatogram of PIK3CD showed c.2314G > A heterozygous mutation in patient 2 and his father had the same site mutation. The arrows indicated the site of PIK3CD gene mutation

Patient 2 was a 4-month-old male that presented with coughing for two days. He had a history of recurrent respiratory tract infections since birth. Complete blood count values were as follows: WBC 10.46 × 10^9^/L (normal, 4–10 × 10^9^/L), Hb 117 g/L (normal, 110–140 g/L), PLT 10 × 10^9^/L (normal, 85–303 × 10^9^/L), and CRP 0.81 mg/L (normal, 0–8 mg/L). Immunological values were as follows: CD3 3749/μl (normal, 4275–5056/μl), CD4 1614/μl (normal, 2008–2695/μl), CD8 1647/μl (normal, 1381–1961/μl), CD4/CD8 1.00 (normal, 1.0–1.9), and CD19 1427/μl (normal, 487–1214/μl). Serum IgG, A, and M levels were 13.31 g/L (normal, 3.22–7.18 g/L), 0.44 g/L (normal, 0.13–0.35 g/L), and 1.96 g/L (normal, 0.23–0.91 g/L), respectively. Serum C3 and C4 were 0.60 g/L (normal, 0.79–1.52 g/L) and 0.01 g/L (normal, 0.16–0.38 g/L), respectively (Table 1). Coombs test was positive, antinuclear antibodies (ANA) were positive (1:1000), and anti-dsDNA showed negative. The EBV and CMV DNA load in the plasma were negative. After treatment with glucocorticoids (1 mg/kg) and immunoglobulin (2 g/kg), significant improvement of thrombocytopenia and respiratory infections were noted after five months. However, over the next year, the patient was hospitalized twice for thrombocytopenia and was given tacrolimus (0.5 g, qd) and mycophenolate mofetil (0.25 g, bid) orally during treatment. Four months ago, the patient was referred to our clinic for thrombocytopenia again. Complete blood count values were as follows: WBC 8.36 × 10^9^/L (normal, 4–10 × 10^9^/L), Hb 112 g/L (normal, 120–140 g/L), PLT 5 × 10^9^/L (normal, 100–300 × 10^9^/L), and CRP 2.33 mg/L (normal, 0–8 mg/L). Serum C3 and C4 were 0.62 g/L (normal, 0.85–1.93 g/L) and 0.08 g/L (normal, 0.12–0.36 g/L), respectively. ANA was positive (1:100) and anti-dsDNA showed positive (+ + +) (Table 1). According to the EULAR/ACR 2019 SLE classification standard, the patient was diagnosed with SLE. The whole exome sequencing of venous blood was performed on the child and his parents. A heterozygous missense mutation c.2314G > A (p.G772S, Fig. 1, Table 2) of PIK3CD gene was revealed in this patient and his father. His father has not any clinical symptoms. His father has the identical mutation to the child. However, there is not any family history of APDS1 or of other immune deficiency (Fig. 2). Subsequently, tacrolimus was discontinued and sirolimus was started. Sirolimus level was maintained approximately 4 ng/mL at a dose of 1 mg/m2. Upon treatment, thrombocytopenia significantly improved and maintained stability. Currently, the patient has shown no signs of complications.

## Discussion and conclusions

We herein report two cases of different variants in PIK3CD gene, presenting with sirolimus relieving hepatosplenomegaly and resistant thrombocytopenia. Our presented case is worth reporting due to the novelty of one of the variants. Besides, the clinical manifestations in two patients with APDS1 are heterogeneous and they are well aligned with known phenotypic data for the disease, which will be reviewed in the following lines.

APDS1 patients typically present with recurrent sinopulmonary infections and increased susceptibility to EBV and/or CMV [9]. In a large pediatric cohort, Almost all APDS1 patients suffered benign lymphoproliferation, including lymphadenopathy, hepatomegaly, splenomegaly or nodular mucosal lymphoid hyperplasia [14]. In accordance with previously studies, recurrent sinopulmonary infections, lymphadenopathy and splenomegaly were the prominent signs in patient 1. Meanwhile, patient 1 had the most frequently reported variant c.3061G > A. The p.E1021K mutation enhances the overactivation of PI3K signaling [15]. The hyperactivation of PI3Kδ plays a crucial role in the pathogenesis of inflammatory and autoimmune diseases. Autoimmune presentations are the features of APDS1 in 28 to 42% of cases [16]. Among these, autoimmune hemolytic anemia (AIHA) and immune thrombocytopenic purpura (ITP) were the most frequent manifestations, followed by Evans syndrome, type 1 diabetes mellitus, enteropathy, arthritis, SLE, autoimmune thyroiditis, sclerosing cholangitis, sjogren syndrome, and autoimmune hepatitis also have been previously reported [17–21]. To date, only a few cases of SLE phenotype have been described in patients with APDS1 [8, 22]. Our patient 2 met classification criteria of SLE due to thrombocytopenia, low level of complement, positive coombs test, and dsDNA. In particular, she had a novel variant c.2314G > A.

A cohort study showed that clinical features of APDS1 patients were incomplete penetrance and highly variable, even in the family carrying the same mutation, ranging from asymptomatic adults to those with primary immunodeficiency, and those with a serious immunodeficiency resulting in early death, to others suffering from lymphoproliferation and malignancy [23]. In accordance with the study, our patient's parents have the same heterozygous missense mutation as their children, but they have not any clinical symptoms. Heurtier L et al. found rare variants c.241G > A and c.371G > A in PIK3CD and confirmed these variants can produce similar phenotypes [24]. Moreover, the novel variants c.1339 + 4G > A and c.2027 + 5C > T have been reported, but no functional assay has been performed [25]. Herein, we have reported a patient with SLE-like phenotype that is in line with the diagnosis of APDS1, who has a novel c.2314G > A variant in PIK3CD gene. Previous studies have suggested that PIK3CD mutations increase PI3Kδ activity resulting in the hyperactivation of the PI3K/AKT/mTOR/S6 signally pathway [26, 27]. Furthermore, studies have indicated that the activity of PI3K and mTOR were implicated in the upregulation of protein S6 phosphorylation induced by IL2 [28]. Several studies have reported that the activation of the PI3K pathway breaks pregerminal center B cell self-tolerance [29]. As shown in the supplement figure, the protein expression levels of pAKT and pS6 were significantly increased (*p* < 0.05, Additional file 1: Fig. S1a, b). Furthermore, elevated expression of IL2 was observed (*p* < 0.01, Additional file 1: Fig. S1c). These results demonstrated hyperactivation of the PI3K/AKT/mTOR/S6 signal pathway. However, no in-depth immune studies have been shown.

The treatment of APDS1 revolves around preventing recurrent infection, reducing lymphoproliferation, and suppressing autoimmunity [30]. In the setting of hypogammaglobulinemia or recurrent infections (even if total IgG is normal), immunoglobulin replacement therapy should be initiated. However, immunoglobulin replacement therapy does not improve autoimmunity and lymphoproliferation. Sirolimus is a mTOR inhibitor that directly targets mTOR and inhibits the PI3K pathway downstream. When sirolimus binds to mTOR it blocks activation of IL-2 induced proliferation of T cells [31]. Currently, sirolimus as the first-line drug for the treatment of APDS1 [5], which is effective in the treatment of lymphocytosis and hepatosplenomegaly [2], but less effective for cytopenia. In a retrospective study, 3 of 14 patients with cytopenia had complete remission with sirolimus and 2 of 14 patients had a partial remission with cytopenia [18, 23]. In addition, 69% of patients did not respond to sirolimus treatment in other studies [32]. Herein, two pediatric patients with APDS1 have received sirolimus treatment after genetic diagnosis. Symptoms, such as hepatosplenomegaly, respiratory infections, and resistant thrombocytopenia, have been effectively controlled, and no obvious adverse reactions have been observed. Leniolisib (CDZ173), an oral p110δ inhibitor, was engineered to decrease PI3Kδ pathway hyperactivity [33]. Currently available studies demonstrated that treatment with leniolisib was well tolerated and reduced lymphadenopathy in patients with APDS1 [34]. In addition to oral mTOR inhibitor or p110δ inhibitor, allogeneic hematopoietic stem cell transplantations (HSCT) is an option for those APDS patients with severe complications and poor responses to currently available conventional therapies [35, 36].

We experienced two patients with APDS1 presenting with diverse clinical phenotypes and different variants in PIK3CD gene. Early genetic diagnosis is essential for providing better treatment for children with APDS1. If whole exome sequencing reveals a variant in PIK3CD gene, sirolimus may be considered to alleviate clinical manifestations such as hepatosplenomegaly and resistant thrombocytopenia. This is a newly report of c.2314G > A variant in PIK3CD gene. Further functional immune studies are needed to explore the mechanisms related to the variant in APDS1.

### Supplementary Information

**Additional file 1: Figure S1.** The protein expression levels of pAKT, pS6 and IL2 in patient 2. Phosphorylation of AKT, S6 and IL2 in patient 2 were significantly higher than in the control group. All data are presented as mean ± SD (n = 3). *p < 0.05, **p < 0.01.

## Data Availability

The original contributions presented in the study are included in the article.

## Abbreviations

PI3K: Activated phosphoinositide3-kinase
APDS1: Activated phosphoinositide3-kinase δ syndrome 1
IEI: Inborn errors of immunity
PI3Kδ: Phosphoinositide3-kinase
SLE: Systemic lupus erythematosus
EBV: Epstein barr virus
CMV: Cytomegalovirus
WBC: White blood cells
Hb: Hemoglobin
PLT: Platelets
CRP: C-reactive protein
ANA: Antinuclear antibodies
AIHA: Autoimmune hemolytic anemia
ITP: Immune thrombocytopenic purpura
HSCT: Allogeneic hematopoietic stem cell transplantations
