# Supplementary figures and images for: Two cases of successful sirolimus treatment for patients with activated phosphoinositide 3-kinase δ syndrome 1
Source: Allergy Asthma Clin Immunol. 2023 Sep 23;19:86. doi: 10.1186/s13223-023-00840-0 (PMC10518115; doi:10.1186/s13223-023-00840-0)

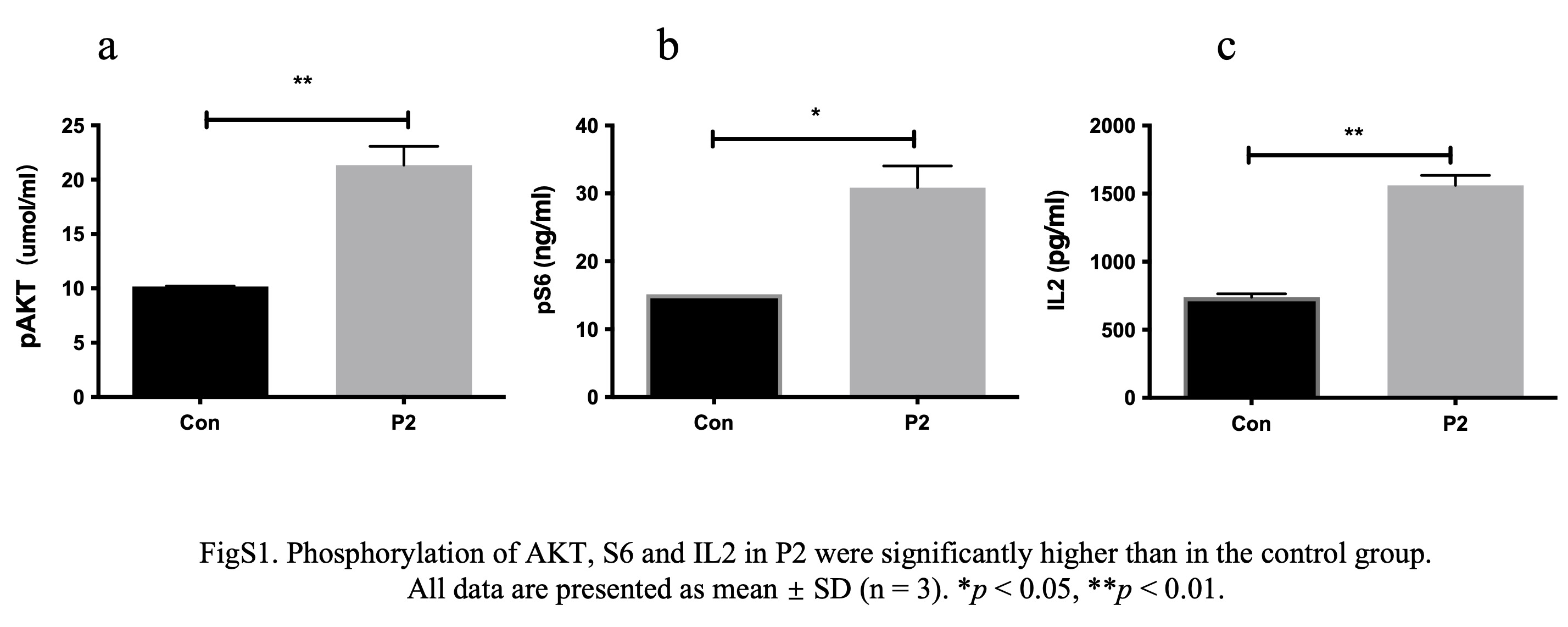

Supplement: Supplementary file 1 — Additional file 1: Figure S1. The protein expression levels of pAKT, pS6 and IL2 in patient 2. Phosphorylation of AKT, S6 and IL2 in patient 2 were significantly higher than in the control group. All data are presented as mean ± SD (n = 3). *p < 0.05, **p < 0.01. [file 13223_2023_840_MOESM1_ESM.jpg]
